# The complete chloroplast genome of *Mahonia oiwakensis* (Berberidaceae), a traditional Chinese medicinal plant

**DOI:** 10.1080/23802359.2020.1714500

**Published:** 2020-01-16

**Authors:** Qunying Xiao, Tu Feng, Yan Yu

**Affiliations:** a School of Ecological Engineering, Key Laboratory of Biological Resources and Ecological Remediation of Guizhou Province, Collaborative Innovation Center of Wetland Eco-engineering of Guizhou Province, Guizhou University of Engineering Science, Bijie, China;; b Key Laboratory of Bio-Resources and EcoEnvironment of Ministry of Education, College of Life Sciences, Sichuan University, Chengdu, China

**Keywords:** Chloroplast genome, *Mahonia oiwakensis*, Berberidaceae

## Abstract

*Mahonia oiwakensis* is a frequently-used traditional Chinese medicinal plant with efficient anti-tumor and anti-inflammatory ability. In this study, we assembled the complete chloroplast (cp) genome of *M. oiwakensis*. The complete cp genome of *M. oiwakensis* is 165,126 bp in length, and has a typical structure with large (LSC 73,382 bp) and small (SSC 18,644 bp) single-copy regions separated by a pair of inverted repeats (IRs 36,550 bp) of large size. The *M. oiwakensis* cp genome contains 148 genes, of which 103 protein-coding genes, 37 tRNA genes, 8 rRNA genes. Phylogenetic analysis shows that *M. oiwakensis* closely clustered with *Mahonia bealei,* but were nested *among* three *speices* of *Berberis*, which robustly supports that *Mahonia* is not monophyletic, thus needs redefinition.

*Mahonia* Nuttall is a taxonomical complex woody genus in Berberidaceae. Some taxonomic treatments (e.g., Lafferriere 1997; Whittemore 1997) merged species of compound leaved (*Mahonia*) with the simple-leaved group (true *Berberis*). Phylogenetic analyses of the chloroplast and nuclear gene implied that *Mahonia* is the non-monophyly (Kim et al. 2004a, 2004b). In the recently published *Flora of China*, *Mahonia* was recognized as a separate genus (Ying et al. 2011).

Many species of the *Mahonia* genus are considered to be medicinal plants (Gancevici 1990; Rohrer et al. 2007). *Mahonia oiwakensis* Hayata (Berberidaceae) is one of traditional Chinese medicinal plants that has been demonstrated to exhibit antioxidant, analgesic, anti-inflammatory and hepatoprotective effects (Chao et al. 2009; Chao et al. 2013; Wong et al. 2009). *M. oiwakensis* is mainly distributed in Eastern and Southern China. Most of the Chinese wild populations of *M. oiwakensis* have been extirpated, probably as a result of over-collecting for medicinal use coupled with other biotic pressures. In this study, we made the first report of a complete plastome for *M. oiwakensis*. The annotated chloroplast genome sequence has been deposited into GenBank with the accession number MN735221.

The mature leaves of *M. oiwakensis* were collected from Daotianhe reservoir, Qixingguan District (105°15′N and 27°18′36″E, altitude 1576 m), Bijie City, Guizhou Province, China and voucher specimens (DTH2017110806) were deposited at BJ (Bijie University Herbarium, Bijie City, Guizhou Province, China). Total genomic DNA was extracted from the silica-dried leaves using the TIANGEN plant genomic DNA extraction kit, following the manufacturer’s instructions. The genomic paired-end (PE150) sequencing was performed on an Illumina Hiseq 2000 instrument (Illumina, San Diego, CA, USA). The complete cp genome was assembled using SOAPdenovo2 (Luo et al. 2012) and the resulting contigs were linked based on overlapping regions after being aligned to *Mahonia bealei* (NC_022457) using Geneious Prime 2020.0.3. Annotation was performed via Geneious Prime 2020.0.3, coupled with manual check and adjustment.

The complete plastome of *M. oiwakensis* is 165,126 bp in length, including two single copy regions (LSC: 73,382 bp and SSC: 18,644 bp) and two inverted repeat regions (IRs: 36,550 bp). The complete chloroplast genome sequence of the *M. oiwakensis* contains a pair of especially large IRs that was also found in *M*. *bealei* (Ma et al. 2013). The whole GC content of the total length, LSC, SSC, and IR regions is 38.1%, 36.4%, 32.4%, and 41.3%, respectively. It contained 148 genes, including 103 protein-coding genes, eight rRNA genes, and 37 tRNA genes were annotated. 33 genes are duplicated in the IR regions, which is congruent with *M*. *bealei* (Ma et al. 2013).

The phylogeny was reconstructed based on 27 Berberidaceae species, using maximum-likelihood (ML). The sequences were aligned using MAFFT v7 (Katoh et al. 2017), and RAxML (v8.2.10) (Stamatakis 2014) were used to construct a maximum likelihood tree. The phylogenetic analysis revealed that *M. oiwakensis* closely clustered with *M. bealei,* but were nested *among* three *speices* of *Berberis* (Figure 1), which robustly supports that *Mahonia* and *Berberis* are not monophyletic. The results are of great implication for the Phylogenetic researches on *Berberis* and *Mahonia* that need redefinition.

**Figure 1.**
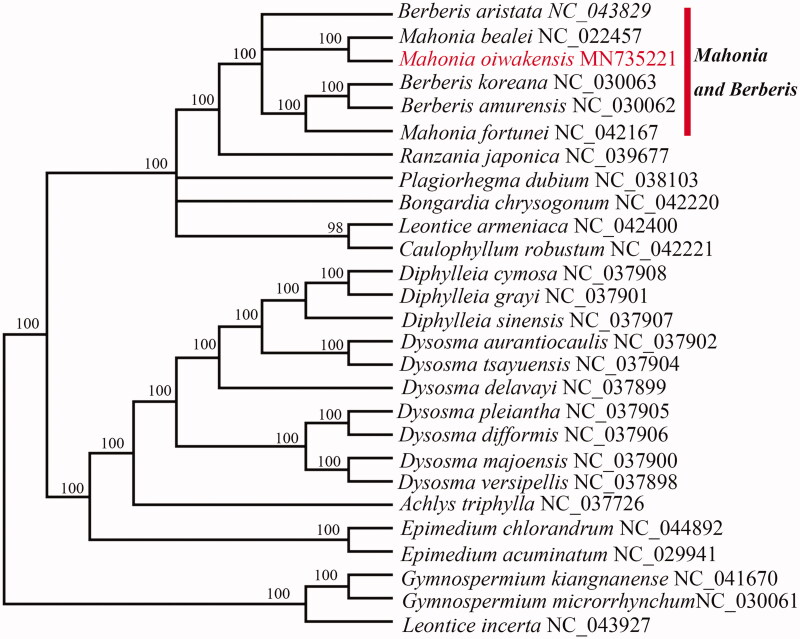
The best ML phylogeny recovered from 27 complete plastome sequences by RAxML. Numbers on the nodes are bootstrap values from 1000 replicates.
